# Successful Kidney Transplantation Despite Ongoing Chronic Norovirus Infection

**DOI:** 10.1016/j.ekir.2023.11.028

**Published:** 2023-12-02

**Authors:** Daan Kremer, Stefan P. Berger, Erik A.M. Verschuuren, Stephan J.L. Bakker, Marjolein Knoester

**Affiliations:** 1 Department of Internal Medicine, Division of Nephrology, University of Groningen, University Medical Center Groningen, Groningen, The Netherlands; 2 Department of Pulmonology and Tuberculosis, Lung Transplantation Program, University of Groningen, University Medical Center Groningen, Groningen, The Netherlands; 3 Department of Medical Microbiology and Infection Prevention, University of Groningen, University Medical Center Groningen, Groningen, The Netherlands

**Keywords:** Caliciviridae, gastroenteritis, immune system, lung transplantation, transplantation, transplant candidacy

## Introduction

Norovirus infection is one of the leading causes of acute gastroenteritis outbreaks worldwide.^1^ Norovirus infections generally present as acute-onset nausea, diarrhea, and abdominal discomfort, and are cleared within 3 days in approximately 85% of cases.^1^ The illness rarely lasts longer than 1 week in immunocompetent patients.^1^ However, immunocompromised patients may develop chronic norovirus infections, suffering from relapsing and remitting episodes of watery diarrhea, unable to clear the virus.^2^ Chronic norovirus infections can last for months, or even years, and can have considerable clinical consequences, including malnutrition, dehydration, alterations in the gastrointestinal barrier, and impaired graft outcome.^3^^,^^4^

The clinical impact of chronic norovirus infections after solid organ transplantation has been described in previous studies.^3^^,^^5^ However, all reported cases concern patients who had undergone a transplant in the past and then encountered a chronic norovirus infection. To our knowledge, there are no reports of patients with an ongoing chronic norovirus infection undergoing a transplantation or retransplantation. Such cases will likely present more frequently in the future, and reporting of such cases is important to understand any potential posttransplant infectious exacerbations and graft outcomes.

In a search for precedents to aid in the decision-making process in such scenarios, we identified a patient in our center who had undergone kidney transplantation while suffering from an ongoing, chronic norovirus infection. This patient is the subject of the current report. Key teaching points of this case are presented in Table 1.

**Table 1:** Teaching points

We report a first case of a safe and successful kidney transplantation in a patient with an ongoing chronic norovirus infection
Key considerations in proceeding with the kidney transplantation, were the patient’s clinical stability despite the ongoing norovirus infection, and the fact that the immunosuppression would remain unaltered after transplantation
This case establishes an important precedent, but we encourage the publication of other cases of transplantations in patients with ongoing norovirus infections, to guide decision-making in such instances. After all, numbers of similar cases will increase, given the increasing numbers of patients receiving second or third organ transplants.

## Case Presentation

The patient was a 63-year old Caucasian female with a history of a bilateral lung transplantation because of lung emphysema. An overview of the patient’s medical history and ongoing medication is provided in Table 2. Four years after lung transplantation, the patient started to experience watery diarrhea and abdominal discomfort. Microbiology analyses showed the presence of fecal norovirus RNA (repeatedly typed by sequencing as norovirus GII.Pg). In the following years, the patient continued to experience gastrointestinal discomfort and episodes of watery diarrhea, for which she intermittently used loperamide. Fecal norovirus loads were monitored and showed ongoing fecal norovirus shedding for 5 years after onset. Absolute lymphocyte count in this period was generally low to normal (mean: 1.2 ± 0.4 billion/l). Despite the ongoing norovirus infection, the patient’s lung graft function and general physical condition remained stable.

**Table 2:** Overview of medical history and medication use prior to the patient’s kidney transplantation

Medical history	Condition	Treatment
Surgical	cholecystitis	cholecystectomy
	diverticulitis	Hartmann procedure
Medical	lung emphysema	Bilateral lung transplantation
		Complication: acute tubular necrosis, several hemodialysis sessions, after which stabilization of kidney function (creatinine ∼160 μmol/l; eGFR ∼25 ml/min per 1.73 m^2^)
	calcineurin inhibitor nephrotoxicity	As a result, progressive kidney disease, culminating in end-stage kidney disease and the decision to opt for preemptive kidney transplantation
Medication use	Medication	Dosage
	darbepoetin alpha	30 μg once per 5 weeks
	azathioprine	50 mg once daily
	calcium carbonate	500 mg once daily
	alphacalcidol	0.25 μg once every 2 days
	loperamide	2 mg once daily
	sodium bicarbonate	1000 mg 3 times daily
	esomeprazole	40 mg once daily
	cotrimoxazole	240 mg once daily
	tacrolimus	1 mg twice daily
	prednisolone	5 mg once daily

eGFR, estimated glomerular filtration rate.

After transplantation, the patient repeatedly had normal IgG levels (>7 g/l) and besides the ongoing chronic norovirus infection, there were no clinical clues for primary immunodeficiency disorders.

**Figure 1:**
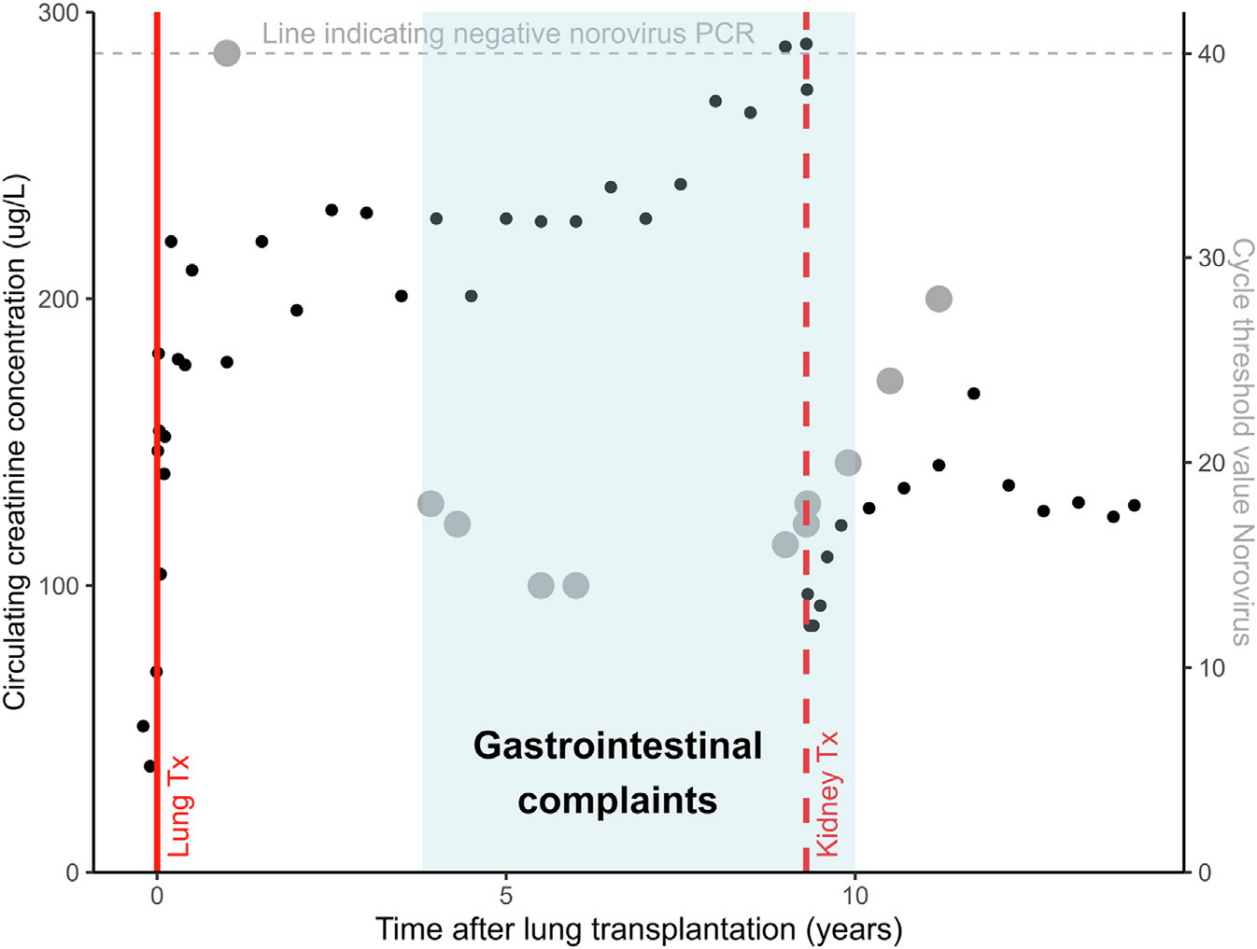
Overview of the trajectory of circulating creatinine concentration (in black) and fecal norovirus cycle threshold value (in gray; lower values indicate higher viral loads) over time. Events of lung transplantation and kidney transplantation are represented by red vertical lines. The period of gastrointestinal complaints is visualized in light blue. All creatinine values are presented in the weeks before and after lung and kidney transplantations, respectively. In other periods, only 1 circulating creatinine concentration measured in the outpatient setting is shown per 6 months during routine outpatient clinic visits to improve clarity.

In the following years, the patient developed kidney failure, which was attributed to posttransplant acute tubular necrosis and calcineurin inhibitor toxicity. The trajectories of creatinine, norovirus, and clinical symptoms are presented visually in Figure 1. The patient was considered for a preemptive kidney transplantation. However, the question was raised about whether the ongoing chronic norovirus infection would be a contraindication. Despite the lack of scientific support for this decision, the clinical team decided that the patient was eligible for kidney transplantation. Key considerations were that the patient had been clinically stable for years, despite her chronic norovirus infection. Moreover, immunosuppressive therapy would remain unaltered after transplantation, because she already used triple-therapy immunosuppression (prednisolone, tacrolimus, and azathioprine) with tacrolimus trough levels that were compatible with those targeted early after kidney transplantation. The planned kidney transplantation was therefore not expected to exacerbate the ongoing norovirus infection. The patient received standard induction therapy following local treatment protocols (including basiliximab 2 hours before and 4 days after transplantation).

The patient successfully underwent a living unrelated kidney transplantation, with excellent graft function immediately after transplantation. Triple immunosuppression was continued with target tacrolimus trough levels between 7 and 9 μg/l, azathioprine, and a prednisolone taper. Measured tacrolimus trough concentrations were similar in the 2 years before kidney transplantation, compared to the 2 years after kidney transplantation (mean 7.4 ± 1.4 μg/l vs. 7.2 ± 1.1 μg/l), and lymphocyte count also remained stable (1.0 ± 0.4 billion/l). Fecal norovirus load remained stable in the first days after transplantation. Clinically, the patient’s stools remained unchanged in the first months after transplantation, without any exacerbations of diarrhea or gastrointestinal discomfort.

Approximately 10 weeks after kidney transplantation (i.e., approximately 9.5 years after lung transplantation), the patient reported that the episodes of diarrhea and gastrointestinal discomfort had stopped completely. In line with this, fecal viral load steadily decreased in the years after transplantation. The episodes of diarrhea and abdominal discomfort remain absent, and kidney and lung graft function are stable to date (i.e., 4 years after kidney transplantation, 13 years after lung transplantation).

## Discussion

To our knowledge, this is the first report of a successful (kidney) transplantation in a patient with an ongoing, chronic norovirus infection. Notably, the prevalence of chronic norovirus infections in organ transplant recipients is substantial, and increasing numbers of patients receive second or third organ transplants.^3^^,^^6^^,^^7^ Therefore, it appears likely that increasing numbers of similar cases of patients with ongoing norovirus infections will be considered for kidney transplantation in the future. This report establishes a precedent that may inform the decision-making process in such scenarios. However, this concerns a single case report which, in itself, is insufficient to infer broader statements on safety in other patients.

Norovirus infections have different clinical characteristics in immunocompromised hosts, compared to immunocompetent hosts. In immunocompromised hosts, such as organ transplant recipients using immunosuppressive therapy, norovirus prevalence is relatively high, with cases presenting year-round rather than the winter-peak that is typically observed in immunocompetent hosts.^2^ Moreover, complications of norovirus infections are more common and have larger clinical impact in immunocompromised hosts. For example, dehydration, malnutrition, and intestinal barrier dysfunction are much more common in immunocompromised hosts; and can compromise graft and patient outcome.^5^ Such severe complications were not observed in our patient, who remained clinically and physically stable during the years of infection.

Treatment options in patients with norovirus infections are primarily supportive, to prevent dehydration. Antimotility agents such as loperamide can be considered, as was done in our patient. Successful treatment of norovirus infections in solid organ transplant recipients using nitazoxanide or immunoglobulins has been reported; however, results are inconsistent.^2^^,^^4^ There are suggestions that temporary discontinuation of proliferation inhibitors may help with norovirus clearance; however, this comes with a risk of eliciting graft rejection.^2^^,^^4^ Interventional trials are needed to robustly evaluate therapeutic treatment options. Meanwhile, supportive care is the only evidence-based treatment.^2^^,^^4^

Literature on the duration of chronic norovirus infections in immunocompromised hosts is limited; however, infections can last indefinitely.^2^ In our case, it stands out that the norovirus fecal load steadily decreased after kidney transplantation, after an ongoing infection with much higher viral loads for years. Although we cannot find reports of chronic norovirus infections with kidney disease being the sole cause, it is widely known that kidney failure is associated with immunological dysfunction.^8^ During kidney failure, both antiinflammatory and proinflammatory cytokines can accumulate due to uremic toxins, oxidative stress, and volume overload.^8^^,^^9^ It appears plausible that, in the reported case, the combination of kidney failure on top of the use of immunosuppressive medication led to the patient’s initial incapacity to control the virus. Improvement of kidney function by the kidney transplantation may have sufficiently improved the patient’s immunological state, to allow her to decrease the viral load after years of infection despite the similar immunosuppressive regimen. However, the actual role of kidney function in the decreasing viral load is strictly hypothetical, because it may also have occurred in absence of the performed kidney transplantation.

In conclusion, this report describes a first case of a safe and successful kidney transplantation in a patient suffering from a chronic norovirus infection. We stress the need for future reporting of similar cases, which can further inform clinicians in the decision-making process of patients with a chronic norovirus infection who are being considered for organ transplantation.

## Disclosure

All the authors declared no competing interests.

## Patient Consent

The authors declare that they have obtained informed consent from the patient discussed in the report.
